# Supplementary figures and images for: JG2: an updated version of the Japanese population-specific reference genome
Source: Hum Genome Var. 2025 Oct 1;12:21. doi: 10.1038/s41439-025-00326-y (PMC12485050; doi:10.1038/s41439-025-00326-y)

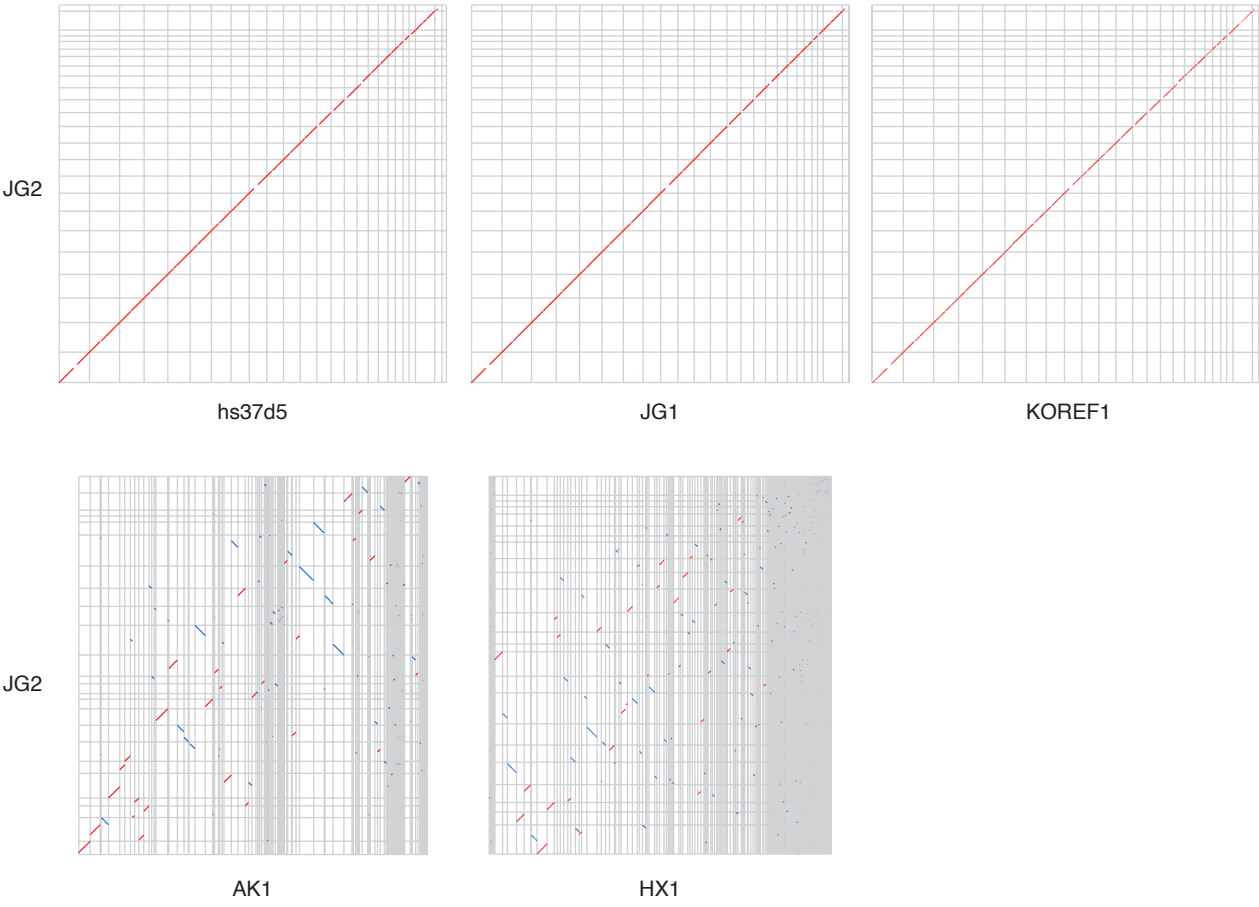

Supplementary Figure 1  
Sriwichain et al

Supplement: Supplementary file 1 — Supplementary Fig. 1 [file 41439_2025_326_MOESM1_ESM.pdf]
