# Supplementary material for: JG2: an updated version of the Japanese population-specific reference genome
Source: Hum Genome Var. 2025 Oct 1;12:21. doi: 10.1038/s41439-025-00326-y (PMC12485050; doi:10.1038/s41439-025-00326-y)
Supplement: Supplementary file 2 — Supplementary Tables [file 41439_2025_326_MOESM2_ESM.pdf]

Supplementary Information

JG2: an updated version of the Japanese population-specific reference genome

Sirawit Sriwichaiin[1], Satoshi Makino[2], Takamitsu Funayama[1,2], Akihito Otsuki[2], Junko Kawashima[2], Yasunobu Okamura[2,3], Shu Tadaka[2], the Tohoku Medical Megabank Project Study Group[2], Fumiki Katsuoka[2,3], Kazuki Kumada[2], Shuichi Tsutsumi[4], Kengo Kinoshita[2,3,5,6], Masayuki Yamamoto[2,7], Gen Tamiya[1,2,8], and Jun Takayama[1,2,8, ✉].

[1]Department of AI and Innovative Medicine, Tohoku University School of Medicine, 2-1, Seiryo-machi, Aoba-ku, Sendai, Miyagi 980-8573, Japan.

[2]Tohoku Medical Megabank Organization, Tohoku University, 2-1, Seiryo-machi, Aoba-ku, Sendai, Miyagi 980-8573, Japan.

[3]Advanced Research Center for Innovations in Next-Generation Medicine, Tohoku University, 2-1 Seiryo-machi, Aoba-ku, Sendai, Miyagi 980-8573, Japan

[4]Genome Science Division, Research Center for Advanced Science and Technology, The University of Tokyo, Tokyo, Japan.

[5]Department of Applied Information Sciences, Graduate School of Information Sciences, Tohoku University, Sendai, Miyagi 980-8579, Japan

[6]Department of In Silico Analyses, Institute of Development, Aging and Cancer (IDAC), Tohoku University, Sendai, Miyagi 980-8575, Japan

[7]Department of Biochemistry and Molecular Biology, Tohoku Medical Megabank Organization, Tohoku University, 2-1 Seiryo-machi, Aoba-ku, Sendai, Miyagi 980-8573, Japan

[8]Statistical Genetics Team, RIKEN Center for Advanced Intelligence Project, Nihonbashi 1-chome Mitsui Building 15F, 1-4-1 Nihonbashi, Chuo-ku, Tokyo 103-0027, Japan.

Correspondence to: Jun Takayama

✉email: jtakayama@tohoku.ac.jp

Department of AI and Innovative Medicine, Tohoku University School of Medicine, 2-1 Seiryo-machi, Aoba-ku, Sendai, Miyagi 980-8573, Japan

Supplementary Table 1. Sequencing platform and sequencing depth using in this study

Platform	jg1a	jg1b	jg1c	Reference
PacBio CLR	122×	123×	128×	(8)
Bionano	123× (BspQI)	160× (DLE-1)	175× (DLE-1)	(8)
	140× (BssSI)			
Hi-C	100.3× (MboI)	70.1× (MboI)	71.3× (MboI)	This study
	49.5× (HindIII)	50.3× (HindIII)	49.2× (HindIII)	
ONT	46.2×	52.0×	43.9×	(8)
mate-pair	13× (dataset1)	12× (dataset 1)	12× (dataset 1)	(8)
	36× (dataset2)	38× (dataset 2)	34× (dataset 2)	This study
paired-end	29× (162 bp)	31× (162 bp)	31× (162 bp)	(8)
	26× (259 bp)	28× (259 bp)	26× (259 bp)	

Depth is calculated by assuming the genome size = 3.0 Gb. CLR, continuous long reads; ONT, Oxford Nanopore Technology.

Supplementary Table 2. Assembly statistics in initial assembly, unzipping, and polishing steps

sample	contig	assembly (2-asm-falcon)			unzipping (3-unzip)			polishing (4-quiver)		
		Total length	N50	Number of contigs	Total length	N50	Number of contigs	Total length	N50	Number of contigs
**jg1a**	primary	2,872,229,904	19,759,866	1,719	2,865,844,877	19,764,383	1,570	2,867,633,543	19,806,459	1,439
	associated/haplotig	126,953,103	41,366	3,096	1,899,278,335	235,067	15,057	1,861,440,548	241,839	13,015
**jg1b**	primary	2,866,558,456	21,053,413	1,663	2,860,907,570	21,047,859	1,526	2,862,109,924	21,088,092	1,386
	associated/haplotig	111,302,058	36,052	3,063	1,847,276,479	220,720	15,203	1,817,898,237	226,102	13,460
**jg1c**	primary	2,863,290,164	18,455,999	1,508	2,857,954,005	18,459,607	1,390	2,859,587,410	18,498,210	1,271
	associated/haplotig	128,014,679	38,090	3,405	1,909,671,674	230,691	15,175	1,877,734,437	235,552	13,289

Supplementary Table 3. Assembly statistics in phase assembly, scaffolding steps

sample	Set	phase	Phasing (5-phase) Total length	N50	Number of contigs	Scaffolding (SALSA2) Total length	N50	Number of contigs	Number of mis-assemblies
jg1a	H→M	0	2,868,973,897	19,816,507	1,423	2,868,216,930	75,737,371	1,058	2,209
		1	2,868,386,599	19,818,126	1,423	2,868,515,945	57,410,013	1,060	2,180
	M→H	0	2,867,671,190	19,827,283	1,423	2,867,793,358	62,217,234	1,039	2,211
		1	2,868,789,306	19,807,350	1,423	2,868,944,936	76,103,143	1,055	2,225
jg1b	H→M	0	2,862,734,228	21,095,395	1,366	2,862,726,396	61,249,395	1,013	2,151
		1	2,862,897,069	21,084,959	1,366	2,862,909,839	63,068,230	1,010	2,216
	M→H	0	2,862,810,851	21,093,630	1,366	2,862,838,203	59,385,241	1,001	2,187
		1	2,862,820,446	21,086,724	1,366	2,862,836,507	63,088,539	1,014	2,168
jg1c	H→M	0	2,860,063,589	18,495,858	1,257	2,860,083,531	62,894,796	926	2,158
		1	2,860,436,381	18,494,518	1,257	2,860,416,655	81,779,830	931	2,118
	M→H	0	2,860,603,511	18,496,741	1,257	2,860,619,566	62,940,103	942	2,141
		1	2,859,896,459	18,493,635	1,257	2,859,882,019	86,925,497	912	2,167

H→M: Phasing with HindIII Hi-C datasets, then scaffolding with MboI Hi-C datasets; M→H: Phasing with MboI Hi-C datasets, then scaffolding with HindIII Hi-C datasets

Supplementary Table 4. Assembly statistics for ONT nanopore reads

sample	assembly stages	number of contigs	total length	NGA50	number of misassemblies
	assembly (Shasta)	4,550	2,834,936,073	10,175,913	1,100
**jg1a**	1st polishing (Racon)	4,550	2,828,673,945	10,241,589	874
	2nd polishing (Medaka)	2,876	2,827,100,153	10,195,383	925
	assembly (Shasta)	7,904	2,839,782,966	9,553,835	1,109
**jg1b**	1st polishing (Racon)	7,904	2,833,862,262	10,218,051	894
	2nd polishing (Medaka)	3,291	2,828,695,661	9,904,179	893
	assembly (Shasta)	6,174	2,832,610,762	9,249,159	897
**jg1c**	1st polishing (Racon)	6,174	2,826,953,599	9,580,139	859
	2nd polishing (Medaka)	2,801	2,824,650,793	9,444,579	818

Supplementary Table 5. Within individual meta-assembly statistics

assembly	jg1a				jg1b				jg1c			
	Misassemblies vs				Misassemblies vs				Misassemblies vs			
	genetic/RH maps	JG1	total length	N50	genetic/RH maps	JG1	total length	N50	genetic/RH maps	JG1	total length	N50
(H.0 + H.1) + (M.0 + M.1)	16	4085	2867177037	75735957	24	4059	2861540671	61247162	14	2816	2858610684	62893376
(H.0 + H.1) + (M.1 + M.0)	16	4072	2867150887	75735957	20	4048	2861619372	61247162	14	2798	2858838197	62893376
(H.0 + M.0) + (H.1 + M.1)	16	4081	2867402314	75735957	20	4061	2861683747	61247162	14	2816	2858730520	62893376
(H.0 + M.0) + (M.1 + H.1)	16	4078	2867463636	75735957	24	4064	2861704097	61247162	14	2809	2858973017	62893376
(H.0 + M.1) + (H.1 + M.0)	16	4059	2867248727	75735957	20	4035	2861529553	61247162	14	2811	2858868283	62893376
(H.0 + M.1) + (M.0 + H.1)	16	4079	2867417997	75735957	24	4036	2861554963	61247162	14	2804	2858771765	62893376
(H.1 + H.0) + (M.0 + M.1)	15	4067	2867279847	57408505	31	3986	2861885057	63067780	17	2768	2859023915	81778283
(H.1 + H.0) + (M.1 + M.0)	15	4069	2867416857	57408505	31	4004	2862124168	63067780	17	2773	2859286998	81778283
(H.1 + M.0) + (H.0 + M.1)	15	4065	2867447452	57408505	31	3979	2861902975	63067780	17	2765	2859162550	81778283
(H.1 + M.0) + (M.1 + H.0)	15	4072	2867671047	57408505	31	3994	2862009415	63067780	17	2769	2859276824	81778283
(H.1 + M.1) + (H.0 + M.0)	15	4067	2867570550	57408505	31	4002	2862074798	63067780	17	2772	2859253014	81778283
(H.1 + M.1) + (M.0 + H.0)	15	4078	2867595906	57408505	31	3994	2861974949	63067780	17	2778	2859440475	81778283
(M.0 + H.0) + (H.1 + M.1)	14	4112	2867094533	62215201	23	4072	2861902602	59379041	17	2820	2859713533	62937813
(M.0 + H.0) + (M.1 + H.1)	14	4096	2866862889	62215201	23	4068	2861867946	59379041	17	2823	2859504686	62937813
(M.0 + H.1) + (H.0 + M.1)	14	4099	2866992474	62215201	23	4068	2861802406	59379041	17	2822	2859404386	62937813
(M.0 + H.1) + (M.1 + H.0)	14	4086	2866840705	62215201	23	4062	2861823335	59379041	26	2816	2859464851	62937813
(M.0 + M.1) + (H.0 + H.1)	14	4096	2866789506	62215201	23	4064	2861818212	59379041	17	2812	2859309823	62937813
(M.0 + M.1) + (H.1 + H.0)	14	4089	2866651407	62215201	23	4055	2861769305	59379041	17	2819	2859563681	62937813
(M.1 + H.0) + (H.1 + M.0)	16	4119	2868019628	76102530	30	4031	2861852855	63087330	25	2779	2858716510	87660285
(M.1 + H.0) + (M.0 + H.1)	16	4114	2868056065	76102530	30	4019	2861841829	63087330	25	2783	2858565596	87638277

(M.1 + H.1) + (H.0 + M.0)	16	4132	2868023531	76102106	30	4024	2862027763	63087330	25	2789	2858854529	87660623
(M.1 + H.1) + (M.0 + H.0)	16	4127	2868036928	76102118	30	4042	2861993783	63087330	16	2794	2858966075	86920416
(M.1 + M.0) + (H.0 + H.1)	16	4127	2867892147	76102118	30	4025	2861902401	63087330	25	2789	2858870290	87638277
(M.1 + M.0) + (H.1 + H.0)	16	4116	2867893559	76102118	30	4027	2861965666	63087330	16	2785	2858947736	86920416

Supplementary Table 6. Selection of pseudo-molecules for each chromosome sequence for JG2

Chromosome	selected meta-scaffold	chromosome	selected meta-scaffold
1	(jg1a + jg1c) + jg1b	13	jg1b + (jg1a + jg1c)
2	jg1a + (jg1c + jg1b)	14	jg1a + (jg1c + jg1b)
3	jg1c + (jg1a + jg1b)	15	jg1a +(jg1b + jg1c)
4	jg1a + (jg1c + jg1b)	16	(jg1c + jg1a) + jg1b
5	(jg1b + jg1a) + jg1c	17	jg1a + (jg1b + jg1c)
6	jg1c + (jg1a + jg1b)	18	jg1c + (jg1b + jg1a)
7	jg1c + (jg1b + jg1a)	19	jg1a + (jg1b + jg1c)
8	jg1b + (jg1c + jg1a)	20	(jg1a + jg1b) + jg1c
9	jg1b + (jg1c + jg1a)	21	(jg1b + jg1c) + jg1a
10	jg1b + (jg1a + jg1c)	22	(jg1c + jg1b) + jg1a
11	(jg1c + jg1b) + jg1a	X	(jg1a + jg1b) + jg1c
12	jg1c + (jg1a + jg1b)	Y	(jg1a + jg1b) + jg1c

Supplementary Table 7. Comparisons between JG2 and other reference genomes

Reference Genome	Misassemblies	Bases align with JG2	Average identity of 1-to-1 alignment (%)
**GRCh38**	581	2824340197 bp (91.11%)	99.79
**T2T-CHM13v2.0**	503	2831216057 bp (90.82%)	99.81
**hs37d5**	803	2826975849 bp (90.10%)	99.79
**AK1**	1637	2792444144 bp (96.15%)	99.84
**KOREF1**	3291	2794514599 bp (87.03%)	99.79
**HX1**	2399	2961895201 bp (94.31%)	99.70
**JG1**	860	2796486111 bp (90.62%)	99.91

Supplementary Table 8. Callable region length

	Callable region (bp)		Genome size (bp)	
chromosome	GRCh38	JG2	GRCh38	JG2
chr1	222,012,800<br>89.2%	215,032,900<br>86.1%	248,956,422	249,784,519
chr2	235,086,000<br>97.1%	231,613,500<br>96.8%	242,193,529	239,194,791
chr3	194,553,300<br>98.1%	193,841,800<br>97.6%	198,295,559	198,550,289
chr4	186,102,300<br>97.8%	184,015,300<br>96.6%	190,214,555	190,509,772
chr5	174,996,500<br>96.4%	174,133,000<br>96.7%	181,538,259	180,090,356
chr6	167,444,700<br>98.0%	166,972,700<br>97.0%	170,805,979	172,157,271
chr7	152,990,600<br>96.0%	149,582,700<br>94.9%	159,345,973	157,618,921
chr8	140,698,900<br>96.9%	140,075,700<br>96.6%	145,138,636	144,985,931
chr9	112,576,800<br>81.3%	107,532,700<br>77.6%	138,394,717	138,512,331
chr10	129,759,500<br>97.0%	127,144,400<br>95.8%	133,797,422	132,738,613
chr11	129,938,900<br>96.2%	128,754,600<br>96.3%	135,086,622	133,713,448
chr12	130,049,400<br>97.6%	129,405,000<br>96.8%	133,275,309	133,726,806
chr13	95,488,700<br>83.5%	94,796,800<br>84.7%	114,364,328	111,966,323
chr14	86,924,100<br>81.2%	86,137,000<br>83.2%	107,043,718	103,507,966
chr15	78,166,700<br>76.6%	77,141,500<br>79.4%	101,991,189	97,152,648
chr16	76,029,900<br>84.2%	72,793,800<br>76.5%	90,338,345	95,206,062
chr17	77,136,300<br>92.6%	75,189,300<br>93.7%	83,257,441	80,226,911
chr18	74,337,000<br>92.5%	73,389,000<br>95.0%	80,373,285	77,264,100
chr19	54,904,300<br>93.7%	54,723,100<br>92.9%	58,617,616	58,932,616
chr20	60,782,600<br>94.3%	58,669,400<br>93.7%	64,444,167	62,634,835
chr21	33,471,800<br>71.7%	32,814,400<br>73.6%	46,709,983	44,565,971
chr22	34,516,000<br>67.9%	32,745,400<br>70.4%	50,818,468	46,508,028
chrX	144,811,600<br>92.8%	144,050,700<br>94.4%	156,040,895	152,668,378
chrY	92,800<br>0.2%	225,700<br>0.5%	57,227,415	50,152,051
